# A Novel Immunomodulatory 27-Gene Signature to Predict Response to Neoadjuvant Immunochemotherapy for Primary Triple-Negative Breast Cancer

**DOI:** 10.3390/cancers13194839

**Published:** 2021-09-28

**Authors:** Toshiaki Iwase, Kim R. M. Blenman, Xiaotong Li, Emily Reisenbichler, Robert Seitz, David Hout, Tyler J. Nielsen, Brock L. Schweitzer, Daniel B. Bailey, Yichao Shen, Xiang Zhang, Lajos Pusztai, Naoto T. Ueno

**Affiliations:** 1 Section of Translational Breast Cancer Research, Department of Breast Medical Oncology, The University of Texas MD Anderson Cancer Center, 1515 Holcombe Blvd, Houston, TX 77030, USA; tiwase@mdanderson.org; 2 Medical Oncology, Yale Cancer Center, 35 Park Street, North Pavilion 1, New Haven, CT 06511, USA; kim.blenman@yale.edu (K.R.M.B.); xiaotong.li@yale.edu (X.L.); 3 Pathology Yale Cancer Center, 35 Park Street, North Pavilion 1, New Haven, CT 06511, USA; emily.reisenbichler@health.slu.edu; 4 Oncocyte Corporation, 15 Cushing, Irvine, CA 92618, USA; rseitz@oncocyte.com (R.S.); dhout@oncocyte.com (D.H.); tnielsen@oncocyte.com (T.J.N.); bschweitzer@oncocyte.com (B.L.S.); dbailey@oncocyte.com (D.B.B.); 5 Molecular and Cellular Biology, Baylor College of Medicine, 1 Baylor Plaza, Houston, TX 77030, USA; yichao.shen@bcm.edu (Y.S.); xiangz@bcm.edu (X.Z.)

**Keywords:** triple-negative breast neoplasms, neoadjuvant therapy, immunotherapy, biomarkers, tumor microenvironment

## Abstract

**Simple Summary:**

Through analysis of specimens from patients with primary triple-negative breast cancer (TNBC) enrolled in a neoadjuvant clinical trial assessing durvalumab with chemotherapy, we confirmed a novel 27-gene immuno-oncology (IO) signature that generates an IO score to predict the pathologic complete response (pCR) of primary TNBC to neoadjuvant immunotherapy with the PD-L1 blocker durvalumab with chemotherapy. Combining the 27-gene IO signature with PD-L1 immunohistochemistry strengthened the model’s predictive power of the pCR. Furthermore, the comprehensive computational analysis revealed that the 27-gene IO signature corresponded with an immunogenic tumor microenvironment.

**Abstract:**

A precise predictive biomarker for TNBC response to immunochemotherapy is urgently needed. We previously established a 27-gene IO signature for TNBC derived from a previously established 101-gene model for classifying TNBC. Here we report a pilot study to assess the performance of a 27-gene IO signature in predicting the pCR of TNBC to preoperative immunochemotherapy. We obtained RNA sequencing data from the primary tumors of 55 patients with TNBC, who received neoadjuvant immunochemotherapy with the PD-L1 blocker durvalumab. We determined the power and accuracy in predicting pCR for the immunomodulatory (IM) subtype identified by the 101-gene model, the 27-gene IO signature, and PD-L1 expression by immunohistochemistry (IHC). The pCR rate was 45% (25/55). The odds ratios for pCR were as follows: IM subtype by 101-gene model, 3.14 (*p* = 0.054); 27-gene IO signature, 4.13 (*p* = 0.012); PD-L1 expression by IHC, 2.63 (*p* = 0.106); 27-gene IO signature in combination with PD-L1 expression by IHC, 6.53 (*p* = 0.003). The 27-gene IO signature has the potential to predict the pCR of primary TNBC to neoadjuvant immunochemotherapy. Further analysis in a large cohort is needed.

## 1. Introduction

Triple-negative breast cancer (TNBC) is the most aggressive molecular subtype of breast cancer. Although patients with TNBC who achieve a pathologic complete response (pCR) after neoadjuvant treatment have a favorable prognosis, those without a pCR have shorter progression-free and overall survival [1]. Recent clinical trials have shown that immune checkpoint inhibitor (ICI) treatment significantly improved the outcomes of patients with TNBC.

To date, several potential predictive biomarkers for ICI treatment have been investigated, including PD-L1 expression by immunohistochemistry (IHC), the number of tumor-infiltrating lymphocytes, T-cell receptor clonality, tumor mutational burden, and tumor microenvironment (TME) features [2]. Of these, the one most utilized in clinical trials for TNBC is PD-L1 expression by IHC [3,4,5,6]. Clinical trials showed that among patients with metastatic TNBC treated with ICI, patients with PD-L1 expression by IHC had significantly longer progression-free survival than patients without PD-L1 expression by IHC [5,6]. However, PD-L1 expression by IHC is not useful to select patients with early-stage TNBC for neoadjuvant ICI treatment because anti-programmed cell death protein 1 (anti-PD-1)/anti-PD-L1 drugs improve pCR rates regardless of PD-L1 status by IHC [3,4]. The latest analysis of the KEYNOTE-522 phase 3 trial showed that the combination of pembrolizumab with chemotherapy in early-stage TNBC patients significantly prolonged an event-free survival (hazard ratio = 0.63; 95% confidence interval (CI), 0.48–0.82: *p* = 0.00031) with higher pCR rate (64.8%) compared to the placebo group, leading to the approval by the U.S. Food and Drug Administration [3,7]. However, the PD-L1 IHC did not show any predictive value in the trial.

There remains a critical need for a predictive biomarker for neoadjuvant ICI treatment in patients with TNBC. Previous work on molecular subtyping of TNBC may offer a way forward in this area. In 2011, Lehmann et al. classified TNBC into six molecular subtypes, including basal-like 1 and 2 (BL1 and BL2), immunomodulatory (IM), mesenchymal (M), mesenchymal stem-like (MSL), and luminal androgen receptor, by clustering 2188 genes from 587 publicly available TNBC cases [8]. After the initial report, Ring et al. recapitulated the 2188-gene model with 101 genes while retaining IM as a modifier, rather than a distinct subtype, because an “IM signature” was observed across the other molecular subtypes [9]. The IM signature is enriched for genes involved in immune cell processes, including immune cell signaling and antigen processing/presentation [8]. Furthermore, the IM signature correlates with a high expression level of immune checkpoint regulatory genes, including *CTLA4*; *CD274*, which encodes PD-L; and *PDCD1*, which encodes PD-1 [10]. Since these immune-related signaling pathways and regulatory genes are critical targets of ICI treatment, we expected that the IM signature would be a predictive biomarker for ICI treatment in patients with TNBC.

To develop a clinically applicable assay to identify likely responders to ICI treatment, we have established a 27-gene immuno-oncology (IO) signature that derived from the 101-gene model [11]. The 27-gene IO signature uses a threshold predefined in independent TNBC datasets to separate strong IM-positive cases from M/MSL-positive cases, which have an inverse correlation to the IM subtype [10]. The advantages of the 27-gene IO signature are the following: (1) the assay can be performed by qPCR with a pre-established threshold and (2) the assay can be performed using mRNA expression data obtained with RNA sequencing or microarrays [11]. The 27-gene IO signature recently predicted survival in lung cancer patients who received ICI treatment [12].

The primary objective of the current study was to determine the accuracy of the 27-gene IO signature in predicting the pCR of TNBC to neoadjuvant immunochemotherapy. We compared the accuracy of the 27-gene IO signature, the IM subtype identified by the 101-gene model, and PD-L1 expression by IHC in predicting pCR of TNBC. We also investigated the accuracy of the combination of the 27-gene IO signature and PD-L1 expression by IHC. Moreover, to understand the TME’s baseline immune landscape in relation to the IM signature and pCR, we performed a comprehensive computational analysis by using an RNA sequencing dataset from the primary tumors.

## 2. Materials and Methods

### 2.1. Design of the Clinical Trial and Sample Acquisition

Baseline core needle biopsy specimens were collected from patients with stage I-III primary TNBC enrolled in a phase I/II trial of immunochemotherapy including PD-L1 blockade with durvalumab (NCT02489448). The trial included patients with newly diagnosed stage I–III TNBC defined by the American Society of Clinical Oncology/College of American Pathologists guidelines: fewer than 10% of tumor cells positive for estrogen receptor, fewer than 10% of tumor cells positive for progesterone receptor, and negative for human epidermal growth factor receptor 2. All patients included in the study participated voluntarily, provided written informed consent, and underwent core needle biopsy and a blood draw for the correlative study.

The treatment regimen of the trial was nab-paclitaxel 100 mg/m^2^ in weeks 1–12, with concurrent durvalumab in weeks 1, 3, 5, 7, 9, and 11; dose-dense anthracycline/cyclophosphamide in weeks 13, 15, 17, and 19; nab-paclitaxel in weeks 14, 16, 18, and 20. During the phase I part of the trial, two dose levels of durvalumab were assessed: 3 mg/kg every 2 weeks and 10 mg/kg every 2 weeks. During the phase II part of the trial, the 10 mg/kg dose was assessed for efficacy. The primary efficacy endpoint was pCR, which was defined as the absence of residual invasive tumor on hematoxylin and eosin evaluation of the breast surgical specimen and all resected regional lymph nodes following completion of neoadjuvant immunochemotherapy (ypT0/Tis, ypN0). The primary safety and efficacy results were published earlier [13].

**Figure 1:**
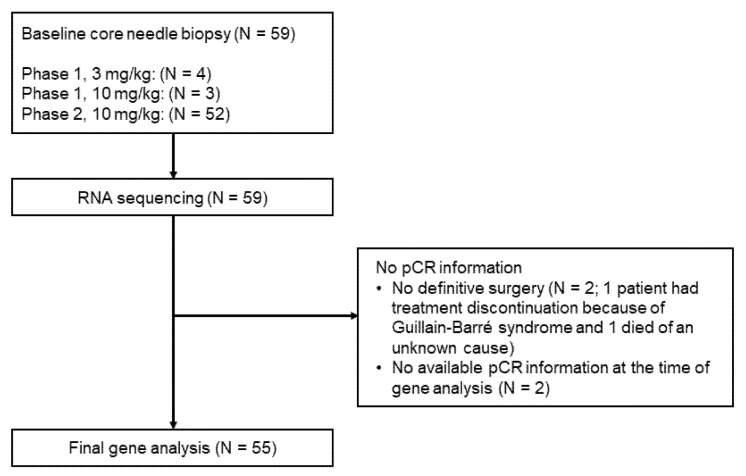
CONSORT diagram of the study. pCR, pathologic complete response.

Between 18 December 2015 and 21 November 2018, 69 patients were screened at the Yale Cancer Center and affiliated regional care centers, and 60 patients consented to participate in the study. One patient withdrew consent before starting the study. The phase I part included seven patients, and the phase II part included 52 patients. Baseline core needle biopsy specimens for the correlative study were obtained from 59 patients. Four patients had no pCR results available, including one patient who discontinued treatment before definitive surgery because of Guillain-Barré syndrome, one patient who died of an unknown cause before definitive surgery, and two additional patients who were not available for pCR information at the time of gene analysis. Those four patients were excluded from the analysis. In total, 55 patients were the subjects of this biomarker analysis (Figure 1).

### 2.2. RNA Sequencing

RNA was extracted from core needle biopsy specimens of the tumor, collected into RNAlater, and stored at −80 °C until RNA extraction with an RNeasy Plus Kit (Qiagen Hilden, Germany). The poly(A)-enriched mRNAs were sequenced on the Illumina platform using NovaSeq paired-end, 100 bp fragments with a depth of 50 million reads at the Yale Center for Genomic Analysis. Quality control of raw read files was performed using FastqQC v0.11.5. Adapter sequences were identified and trimmed using Trimmomatic v0.36. Sequencing reads were aligned against the human genome (hg38) with STAR v2.5.3a using two-pass mode and default parameters. The alignment quality and strandness were checked using RSeQC v2.6.4. Gene expression was quantified using RSEM v1.3.0 with an option for strand-specific library preparation protocol. ENSEMBL release 91 was used to annotate reads with human genes. Only genes with expression in at least one patient were included in further analysis.

### 2.3. Evaluation of the 27-Gene IO Signature

Table 1 shows all the genes in the 27-gene IO signature. We derived the 27-gene IO signature from the previously described 101-gene model [9], guided by the insight that the IM, M, and MSL subtypes would provide information about the TME [10]. The 27 genes in the 27-gene IO signature were the ones most correlated to the IM, M, and MSL subtypes of the 101-gene model [11]. TNBC specimens were isolated from datasets obtained by the Gene Expression Omnibus [9]. A threshold for positivity was determined using these TNBC specimens but set using the area under the curve rather than the significance of correlation [9]. For the present study, we compared the predictive accuracy of the 27-gene IO signature and the 101-gene model. The clinical endpoint was the odds ratios (ORs) for pCR. We further assessed the predictive power of the 27-gene IO signature using standard measures of diagnostic discrimination.

To evaluate whether chemotherapy confounds the predictive accuracy of the 27-gene IO signature, we examined the basal subtypes (BL1 and BL2) determined by the 101-gene model. The BL1 subtype exhibits a significantly higher pCR rate than the BL2 subtype to neoadjuvant chemotherapy [14]. Therefore, we evaluated the confounding effect of chemotherapy by investigating how the predictive power of the 27-gene IO signature and 101-gene model differed between patients with the BL1 or BL2 subtype compared to patients with other subtypes.

### 2.4. Evaluation of PD-L1 Expression by IHC

PD-L1 IHC was performed on 5 μm formalin-fixed, paraffin-embedded samples of baseline core needle biopsy specimens using the VENTANA PD-L1 SP263 antibody and Ventana Benchmark autostainer (Roche, Basel, Switzerland) following the manufacturer’s instructions. IHC with the VENTANA PD-L1 SP263 antibody was selected because it is the companion diagnostic test that is being developed together with durvalumab. Scoring was performed by two pathologists at the Yale Cancer Center. Two levels of PD-L1 positivity were assessed: (1) expression in at least 1% of the tumor and/or immune cells, and (2) expression in more than 50% of tumor cells and/or more than 10% of immune cells.

### 2.5. Exploration of the Baseline Immune Landscape of the TME

Previously, we used a quantitative fluorescence assay (AQUA) to investigate the differences between patients with and without a pCR in the number of infiltrating CD8+ T cells, the number of CD68+ cells, and quantitative PD-L1 expression on tumor cells, CD68+ cells, and stromal cells in the same samples. We found no difference in the amount of CD68+ cells in the tumor or stromal compartments between patients with and without a pCR, but PD-L1 expression in tumor cells, in CD68+ cells, and in the stroma was significantly higher in patients with a pCR than in those without a pCR [13]. To further explore the baseline tumor immune landscape and cell types associated with pCR and the 27-gene IO signature, we estimated the immune cell composition of the biopsy specimens using the TIMER (Tumor IMmune Estimation Resource), CIBERSORT (Cell-type Identification By Estimating Relative Subsets Of RNA Transcripts), and xCell algorithms [15,16,17].

### 2.6. Statistical Considerations

With pCR (ypT0, ypN0) as a primary endpoint, a binary logistic regression model was applied to generate ORs with 95% CI to determine the predictive accuracy of the 27-gene IO signature (primary objective), the 101-gene model, and PD-L1 expression by IHC. The predictive accuracy of the 27-gene IO signature, the 101-gene model, and PD-L1 expression by IHC was determined using diagnostic indicators, including sensitivity, specificity, positive likelihood ratio (PLR), negative likelihood ratio (NLR), positive predictive value (PPV), and negative predictive value (NPV). For the 27-gene IO signature, we used the 0.09 threshold prospectively established during the development of the algorithm and previously validated in lung cancer as associated with response to ICI treatment [11]. For the 101-gene model, the threshold of 0.195 was previously selected for the IM subtype [9]. However, this threshold was chosen to be conservative in the absence of a clinical standard; therefore, we also explored thresholds of 0.17 and 0.10. We used R and RStudio for the statistical analysis (R 3.6.1, R Foundation). All *p* values were two-sided, and *p* less than 0.05 was defined as statistically significant. The various immune markers that we assessed are not independent measurements; gene memberships overlap. Therefore, only nominal *p* values are reported without adjustment for the multiple comparisons.

## 3. Results

### 3.1. Predictive Power of the 101-Gene Model and 27-Gene IO Signature

Of the 55 patients in the analysis, 25 (45%) achieved a pCR (Table 2). The logistic regression models on pCR as a continuous value showed significant predictive power of the 101-gene model (OR, 24.15; 95% CI, 3.17–259; *p* = 0.0015) and of the 27-gene IO signature (OR, 16.42; 95% CI, 3.61–98.2; *p* < 0.001). The logistic regression models for the 101-gene model with three prespecified alternative IM subtype thresholds only approached significant predictive power (OR, 3.03–3.14; *p* = 0.054), and results were very similar with all three thresholds (Table 3). However, the logistic regression model with the predefined threshold for likely response with the 27-gene IO signature, the prespecified primary objective, showed significant predictive power (OR, 4.13; 95% CI, 1.36–13.5; *p* = 0.012). Additionally, the 27-gene IO signature had a higher PLR (2.09) and lower NLR (0.51) than the 101-gene model.

### 3.2. Predictive Accuracy of the PD-L1 IHC Results and the 27-Gene IO Signature

**Figure 2:**
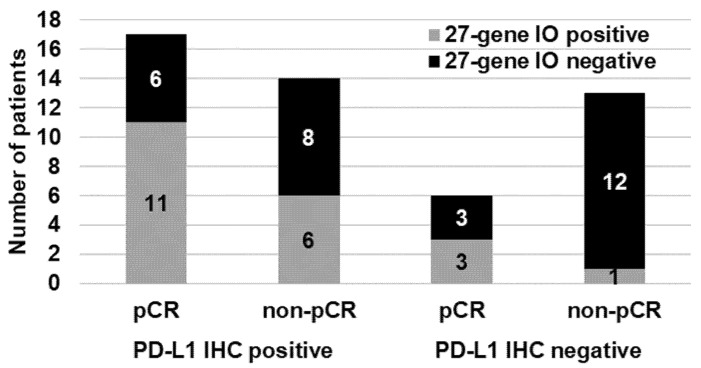
Distributions of patients by pCR status and 27-gene IO signature status in the 31 patients with and 19 patients without PD-L1 expression by IHC. PD-L1 IHC was performed by using the VENTANA PD-L1 SP263 antibody.

Next, we compared the predictive accuracy of the PD-L1 IHC results and the 27-gene IO signature. High-quality PD-L1 IHC results were available for 50 patients (Figure 2). Among the 31 (62%) patients with positive PD-L1 IHC results (expression in ≥1% of tumor and/or immune cells), 17 (55%) had a pCR. Among the 19 (38%) patients with negative PD-L1 IHC results, 6 (32%) had a pCR. Three of six (50%) PD-L1-negative patients with a pCR but only 1 of 13 (8%) PD-L1-negative patients without a pCR had TNBC positive for the 27-gene IO signature (Figure 2). The PPV, NPV, PLR, and NLR for PD-L1 expression by IHC were 0.55, 0.68, 1.43, and 0.54, respectively; the corresponding values for the 27-gene IO signature were 0.60, 0.73, 2.09, and 0.51, respectively (Table 3).

We then tested the combination of the 27-gene IO signature and PD-L1 expression by IHC to determine if the combination had better predictive accuracy. For patients who either had positive PD-L1 IHC results (expression in ≥1% of tumor and/or immune cells) or had TNBC positive for the 27-gene IO signature, the PPV, NPV, PLR, and NLR were 0.57, 0.80, 1.57, and 0.29, respectively, and the OR was higher than the OR for either single test (OR, 5.33; 95% CI, 1.27–22.32; *p* = 0.022) (Table 3). For patients with either a high level of PD-L1 positivity (expression in >50% of tumor cells and/or >10% of immune cells) or TNBC positive for the 27-gene IO signature, the PPV, NPV, PLR, and NLR were 0.68, 0.72, 2.53, and 0.47, respectively, and the OR was higher than for any other subtyping method or combination (OR, 6.53; 95% CI, 1.90–22.50; *p* = 0.003).

### 3.3. Predictive Power of the 27-Gene IO Signature in the BL1 and BL2 Groups

Of the 55 patients in the analysis, 26 had either the BL1 subtype (*n* = 18) or the BL2 subtype (*n* = 8) as determined by the 101-gene model. Ten (56%) of the patients with the BL1 subtype and three (38%) of those with the BL2 subtype had a pCR. However, neither subtype was a significant independent predictor of pCR (data not shown). As noted above, the OR for pCR among patients with TNBC positive for the 27-gene IO signature was 4.13 (*p* = 0.012). Interestingly, among the 26 patients with either BL1 or BL2 subtype, positivity for the 27-gene IO signature was not a significant predictor of pCR (OR, 1.87; 95% CI, 0.39–8.9; *p* = 0.43), but among the 29 patients with neither BL1 nor BL2 subtype, positivity for the 27-gene signature was a strong predictor of pCR (OR, 9.33; 95% CI, 1.65–52.68; *p* < 0.00114).

### 3.4. Tumor Immune Landscape in IM Signature and Non-IM Signature by Deconvolution

We used TIMER (Figures S1 and S2), CIBERSORT (Figures S3 and S4), and xCell (Figures S5 and S6) to computationally explore estimated fractions of immune cell subtypes based on mRNA expression patterns.

#### CD8+ and CD4+ T Cells

Overall, computational methods demonstrated a higher presence of CD8+ T cells and CD4+ T cells in patients with TNBC positive for the 27-gene IO signature and in patients with a pCR (Table 4 and Table 5).

For CD8+ T cells, CIBERSORT and xCell showed significantly higher levels of CD8+ T cells in patients with TNBC positive for the 27-gene IO signature than in patients with TNBC negative for this signature. Similarly, xCell showed significantly higher levels of CD8+ T cells in patients with pCR than in those with non-pCR; this difference was marginally significant according to CIBERSORT. However, TIMER showed no significant differences in levels of CD8+ T cells by 27-gene IO signature or pCR status.

For CD4+ T cells, TIMER showed significantly or marginally significantly higher levels of CD4+ T cells in patients with TNBC positive for the 27-gene IO signature than in patients with TNBC negative for this signature and in patients with pCR than in those with non-pCR. CIBERSORT showed that pCR cases tended to have a higher presence of resting memory CD4+ T cells, activated memory CD4+ T cells, and follicular helper T cells. By CIBERSORT, patients with TNBC positive for the 27-gene IO signature had a significantly higher calculated presence of activated memory CD4+ T cells.

## 4. Discussion

The present study clinically confirmed the IM signature as a significant biomarker of response of TNBC to neoadjuvant immunochemotherapy. Our 27-gene IO signature demonstrated better accuracy than PD-L1 positivity by IHC according to the SP263 assay as a predictive biomarker for pCR. Combining the 27-gene IO signature with PD-L1 positivity by IHC further improved the predictive accuracy. Patients with negative results for both PD-L1 expression by IHC and the 27-gene IO signature had a low probability of pCR (20% (3/15)). This is the first report defining a potential predictability of pCR with the IO signature.

PD-L1 expression by IHC has been widely applied as a predictive biomarker in immunotherapy clinical trials with PD-1/PD-L1 inhibitors. For ICI treatment in metastatic TNBC, PD-L1 positivity by IHC on tumor-infiltrating immune cells or tumor cells correlated with prolonged survival after treatment [5,6]. The advantages of PD-L1 IHC include its ready availability through pathology departments that are already familiar with IHC-based assays and the strong clinical trial data that support the clinical utility of IHC-based assays in metastatic TNBC. The major drawbacks of PD-L1 IHC are the variability of staining results across pathologists, a large number of different assays, and scoring methods that differ from drug to drug and cancer type to cancer type [18]. A standardized assay uniformly applicable across cancer types and different ICI agents that retain the clinical utility of the various FDA-approved IHC assays would be welcomed by the medical community.

In the KEYNOTE-522 study, the PD-L1 positivity did not clearly show a clinical benefit of immunochemotherapy for pCR or event-free survival compared to those tumors with PD-L1 negativity. Whereas in metastatic TNBC, PD-L1 protein expression is a prerequisite for experiencing benefit from ICI treatment, in early-stage TNBC, no marker exists to select patients for neoadjuvant ICI treatment. The present study demonstrated that the predictive power of the standardized a priori-defined 27-gene IO signature was superior to the predictive power of PD-L1 expression by the SP263 IHC assay as reflected by a higher OR and greater PPV. Of note, the combination of the 27-gene IO signature and PD-L1 expression by IHC had greater predictive power than either alone, and the OR was maximized when the 27-gene IO signature was combined with high PD-L1 positivity, defined as PD-L1 expression in more than 50% of tumor cells and/or more than 10% of immune cells. This result suggests that the 27-gene IO signature could be useful to further refine the predictive function of PD-L1 IHC results. However, a validation study will be needed to answer the critical question if the 27-gene IO signature alone or in combination with PD-L1 IHC results can identify patients with early-stage TNBC who have a low probability of benefit from the addition of ICI treatment to neoadjuvant chemotherapy.

In the case of the IM subtype, a previous analysis of 583 TNBC tumors showed that the IM subtype was significantly positively correlated with the expression of immuno-regulatory genes such as T cell and B cell markers and chemokines (*IDO1*, *CTLA4*, *CD274*, *FOXP3*, *CXCL9*, *CD8A*, *CCL5*, *CXCL13*, *IGKC*, *CD80*, and *CR2*) [10]. Another study showed that tumors with the IM signature had a higher expression level of the immune checkpoint regulatory genes *CD274* and *PDCD1*, which encode PD-L1 and PD-1, respectively [19]. These results support the biological robustness of using a high expression of IM genes as a molecular predictive marker for ICI treatment. We further strengthened the 27-gene IO signature by adding the genes in the M and MSL subtypes, which inversely correlated with the IM subtype. Moreover, in addition to the calculation of the 27-gene IO signature using whole transcriptome RNAseq data demonstrated here, the signature has been translated to a 27-gene RT-qPCR, allowing the adoption of the 27-gene IO signature with PD-L1 expression by IHC in the clinic on a technically simple and cost-effective platform.

We investigated the impact of the BL1 and BL2 subtypes on the response to immunochemotherapy to estimate how chemotherapy affected the predictive power of the 27-gene IO signature. In our previous study, we found that TNBC patients with the BL1 subtype had the highest pCR rate (52%) to chemotherapy alone, while those with the BL2 subtype had the lowest pCR rate (0%) [14]. The present analysis also showed that patients with the BL1 subtype had a higher pCR rate (56%) than those with the BL2 subtype (38%). The OR for pCR for patients with TNBC positive for the 27-gene IO signature was approximately 2 for patients with the BL1 or BL2 subtype and greater than 9 for patients with other subtypes. This indicates that the BL subtypes confounded the pCR predictive power of the 27-gene IO signature. In TNBC subtypes other than BL1 or BL2, the 27-gene IO signature remained a strong predictor of response to immunochemotherapy.

The three different computational methods designed to quantitatively estimate the relative proportions of various immune cell types revealed differences in the immune TME between cancers with and without a pCR and positive versus negative for the 27-gene IO signature. In general, cancers with a pCR were positive for the 27-gene IO signature and tended to have higher proportions of CD8+ T cells, CD4+ T cells, proinflammatory M1 macrophages, and dendritic cells, consistent with an immune-activated TME. Activated CD8+ cytotoxic T cells play a central role in antitumor immunity via secreting perforin and granzymes [20]. In the KEYNOTE-001 trial, increased density of CD8+ T cells at the invasive margin or within the tumor parenchyma in the pretreatment samples was significantly associated with response to single-agent pembrolizumab in patients with melanoma [21]. CIBERSORT showed increased CD4+ helper T cells in pCR cases and CD4+ memory T cells in 27-gene-IO-signature-positive cases, while no significant differences were observed for Tregs. CD4+ T cells play a key role in activating cytotoxic CD8+ T cells via secreting IL-2 and modulating activated dendritic cells [22]. Activated dendritic cells function as antigen-presenting cells and activate cytotoxic CD8+ T cells via presenting tumor-associated antigens on MHC class I molecules to the T cell receptor [23,24]. We observed a trend for increased dendritic cells in the pCR and 27-gene-IO-signature-positive cases consistent with the role of dendritic cells in activating CD8+ T cells. Overall, these results show that the 27-gene IO signature correlated with an immunogenic TME, and the immune-active TME status was associated with pCR.

Our study has several limitations. First, the expression of the 27 genes was determined from RNA sequence data, not from the RT-qPCR assay that is being developed for commercial use. We applied the same positivity threshold and gene coefficients to the 27-gene IO signature in the present study as used in the RT-qPCR assay and an earlier validation study in lung cancer. Second, we could not perform a detailed analysis regarding the association between clinicopathological features, pCR, and 27-gene IO because of the limited information. Although the analysis with available information did not provide any significant results (T stage vs. pCR, *p* = 0.413; T stage vs. 27-gene IO signature positive, *p* = 0.855; N stage vs. pCR, *p* = 0.655; N stage vs. 27-gene IO signature positive, *p* = 0.696). More detailed analysis with sufficient clinicopathological features would be needed. Third, the core needle biopsy specimens used in this study may not fully represent the broader immune TME of a given cancer. However, this is unlikely to be a major confounder because we previously demonstrated that multiple different core biopsy specimens obtained from different segments of breast cancers yielded highly concordant estimates of immune cell composition, indicating that the immune TME in a single biopsy specimen is likely to be representative of the immune TME in the entire tumor [25]. We also recognize the limitations of estimating immune cell components based on gene expression data. We observed substantial heterogeneity in the results generated by the different immune cell deconvolution algorithms due to the different assumptions these algorithms make and the overlap in gene expression profiles between different immune cell types. In the absence of histologic confirmation, the computationally derived immune cellularity estimates are only hypothesis-generating. Most importantly, to demonstrate that the 27-gene IO signature can identify patients who selectively benefit from the inclusion of ICI therapy in their treatment will require studying tissues from randomized trials.

## 5. Conclusions

In summary, we demonstrated statistically significant pCR predictive power for the 27-gene IO signature in patients with primary TNBC who underwent neoadjuvant immunochemotherapy. The predictive performance was superior to that of the previously established 101-gene model and a standard PD-L1 IHC test. The combination of the 27-gene IO signature with the PD-L1 IHC test strengthened the model’s predictive performance. The exploratory computational analysis of the baseline TME revealed that the patients with pCR who had TNBC positive for the 27-gene IO signature tended to have a more immunogenic TME than patients who had TNBC negative for the 27-gene IO signature. A validation study using the tissue samples from randomized studies, especially KEYNOTE-522, will be needed to determine if the 27-gene IO signature alone or in combination with PD-L1 IHC test can identify patients with early-stage TNBC who have a low probability of benefit from the addition of ICI treatment to neoadjuvant chemotherapy.

## Figures and Tables

**Table 1:** Genes included in the 27-gene IO signature.

*APOD*	*GZMB*	*PSMB9*
*ASPN*	*HTRA1*	*PTGDS*
*CCL5*	*IDO1*	*RARRES3*
*CD52*	*IL23A*	*RTP4*
*COL2A1*	*ITM2A*	*S100A8*
*CXCL11*	*KMO*	*SFRP1*
*CXCL13*	*KRT16*	*SPTLC2*
*DUSP5*	*KYNU*	*TNFAIP8*
*FOXC1*	*MIA*	*TNFSF10*

**Table 2:** Patient characteristics.

Characteristic	Number of Patients (%)
All patients	55 (100)
Age, years	
≤40	11 (20)
41–50	18 (33)
51–69	26 (47)
Clinical tumor status	
cT1	18 (33)
cT2	29 (53)
cT3	7 (13)
Unknown	1 (2)
Clinical nodal status	
cN0	28 (51)
cN1	22 (40)
cN2	1 (2)
cN3	3 (5)
Unknown	1 (2)
pCR	
Yes	25 (45)
No	30 (55)

**Table 3:** Predictive accuracy of the original 101-gene model, the 27-gene IO signature, and PD-L1 expression by IHC.

Subtyping Method	Threshold	OR (95% CI)	*p*	Sensitivity (%)	Specificity (%)	PPV	NPV	PLR	NLR
101-gene model	0.17	3.14 (0.98–10.9)	0.054	64.7	63.2	0.44	0.80	1.76	0.56
	0.195	3.14 (0.98–10.9)	0.054	64.7	63.2	0.44	0.80	1.76	0.56
	0.10	3.03 (0.98–10)	0.054	63.2	63.9	0.48	0.77	1.75	0.58
27-gene IO signature	0.09	4.13 (1.36–13.5)	0.012	65.2	68.8	0.60	0.73	2.09	0.51
PD-L1 IHC	PD-L1: 1%	2.63 (0.82–9.21)	0.106	73.9	48.1	0.55	0.68	1.43	0.54
PD-L1+ or 27-gene IO signature	PD-L1: 1%	5.33 (1.27–22.32)	0.022	87	44.4	0.57	0.80	1.57	0.29
PD-L1 high or 27-gene IO signature	PD-L1: >50% in tumor and/or >10% in immune cells	6.53 (1.9–22.5)	0.003	65.4	74.2	0.68	0.72	2.53	0.47

Abbreviations: OR, odds ratio; CI, confidence interval; PPV, positive predictive value; NPV, negative predictive value; PLR, positive likelihood ratio; NLR, negative likelihood ratio.

**Table 4:** Tumor immune landscape in patients with TNBC positive versus negative for the 27-gene IO signature by deconvolution.

Variation		Pos vs. Neg for 27-Gene IO Signature								
		TIMER			CIBERSORT			xCell		
Immune Cell	Subpopulation	Neg (*n* = 32)	Pos (*n* = 23)	*p*	Neg (*n* = 32)	Pos (*n* = 23)	*p*	Neg (*n* = 32)	Pos (*n* = 23)	*p*
CD8+ T cell				0.43	Low	High	0.0072	Low	High	5.10 × 10^−5^
CD4+ T cell	General	Low	High	0.015				Low	High	0.00071
	Resting memory T cell						0.27			
	Activated memory T cell				Low	High	1.80 × 10^−6^			
	Follicular helper T cell						0.5			
	Regulatory T cell						0.52			
Macrophage	General	High	Low	0.029				Low	High	0.00041
	M1				Low	High	9.00 × 10^−5^			
	M2						0.19			
Dendritic cell	General	Low	High	8.00 × 10^−5^				Low	High	1.10 × 10^−5^
	Resting dendritic cell						0.24			
	Activated dendritic cell						0.71			

**Table 5:** Tumor immune landscape in patients with pCR versus non-pCR by deconvolution.

Variation		pCR vs. Non-pCR								
		TIMER			CIBERSORT			xCell		
Immune Cell	Subpopulation	Non-pCR (*n* = 30)	pCR (*n* = 25)	*p*	Non-pCR (*n* = 30)	pCR (*n* = 25)	*p*	Non-pCR (*n* = 30)	pCR (*n* = 25)	*p*
CD8+ T cell				0.67	Low	High	0.089	Low	High	0.036
CD4+ T cell	General	Low	High	0.019						0.22
	Resting memory T cell						0.14			
	Activated memory T cell						0.12			
	Follicular helper T cell				mLow	mHigh	0.072			
	Regulatory T cell						0.64			
Macrophage	General	mHigh	mLow	0.077						0.79
	M1				Low	High	0.0018			
	M2						0.16			
Dendritic cell	General	mLow	mHigh	0.1				mLow	mHigh	0.048
	Resting dendritic cell						0.33			
	Activated dendritic cell						0.6			

Abbreviations: mHigh, marginally high; mLow, marginally low.

## Data Availability

The datasets generated during and/or analyzed in the present study are not publicly available because of commercial confidentiality. However, they are available from the corresponding author upon reasonable request.

## Supplementary Materials

The following are available online at https://www.mdpi.com/article/10.3390/cancers13194839/s1, Figure S1: Gene expression of immune cell populations in the tumor microenvironment in patients with primary triple-negative breast cancer positive (immunomodulatory; IM) and negative (non-IM) for the 27-gene IO signature by TIMER algorithm, Figure S2: Gene expression of immune cell populations in the tumor microenvironment in patients with a pathologic complete response (pCR) or without a pCR (non-pCR) by TIMER algorithm, Figure S3: Gene expression of immune cell populations in the tumor microenvironment in patients with primary triple-negative breast cancer positive (immunomodulatory; IM) and negative (non-IM) for the 27-gene IO signature by CIBERSORT algorithm, Figure S4: Gene expression of immune cell populations in the tumor microenvironment in patients with a pathologic complete response (pCR) or without a pCR (non-pCR) by CIBERSORT algorithm, Figure S5: Gene expression of immune cell populations in the tumor microenvironment in patients with primary triple-negative breast cancer positive (immunomodulatory; IM) and negative (non-IM) for the 27-gene IO signature by xCell algorithm, Figure S6: Gene expression of immune cell populations in the tumor microenvironment in patients with a pathologic complete response (pCR) or without a pCR (non-pCR) by xCell algorithm.
