# Supplementary material for: A Novel Immunomodulatory 27-Gene Signature to Predict Response to Neoadjuvant Immunochemotherapy for Primary Triple-Negative Breast Cancer
Source: Cancers (Basel). 2021 Sep 28;13(19):4839. doi: 10.3390/cancers13194839 (PMC8508147; doi:10.3390/cancers13194839)
Supplement: Supplementary file 1 [file cancers-13-04839-s001.zip › cancers-1373685-supplementary.pdf]

cancers MDPI

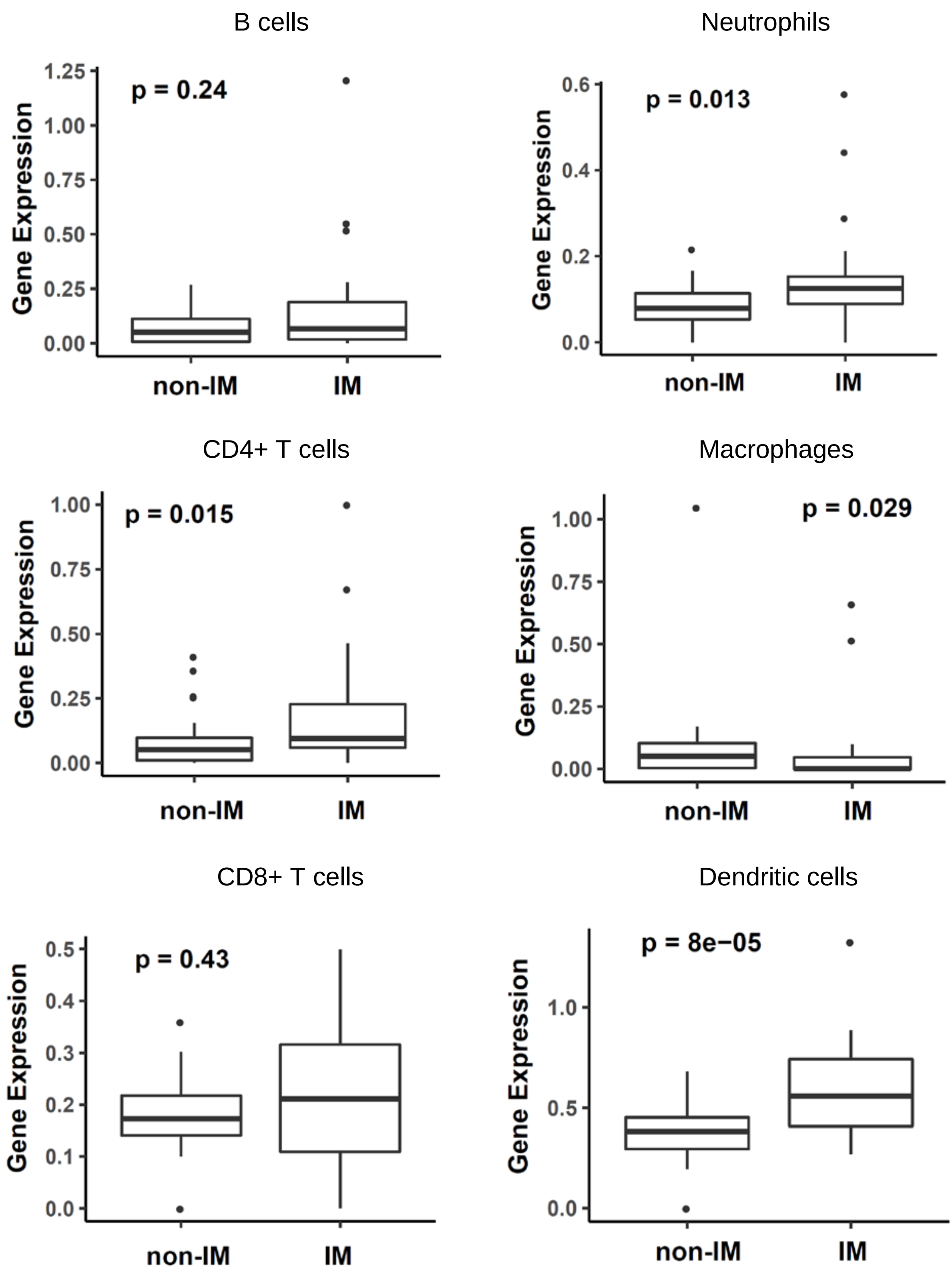

Figure S1. Gene expression of immune cell populations in the tumor microenvironment in patients with primary triple-negative breast cancer positive (immunomodulatory; IM) and negative (non-IM) for the 27-gene IO signature by TIMER algorithm. Boxes indicate 25th percentile, median, and 75th percentile. Each dot corresponds to one patient.

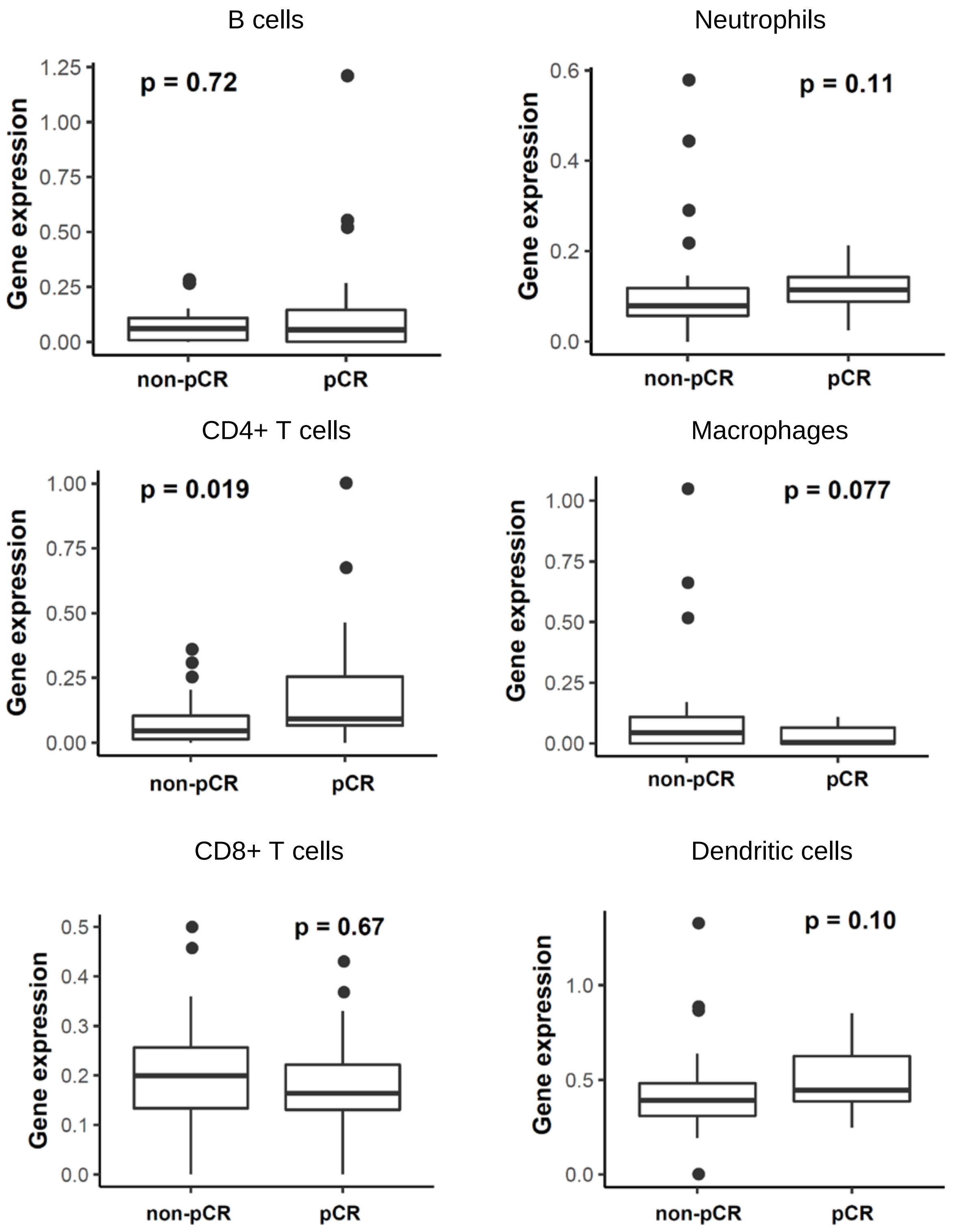

Figure S2.. Gene expression of immune cell populations in the tumor microenvironment in patients with a pathological complete response (pCR) or without a pCR (non-pCR) by TIMER algorithm.

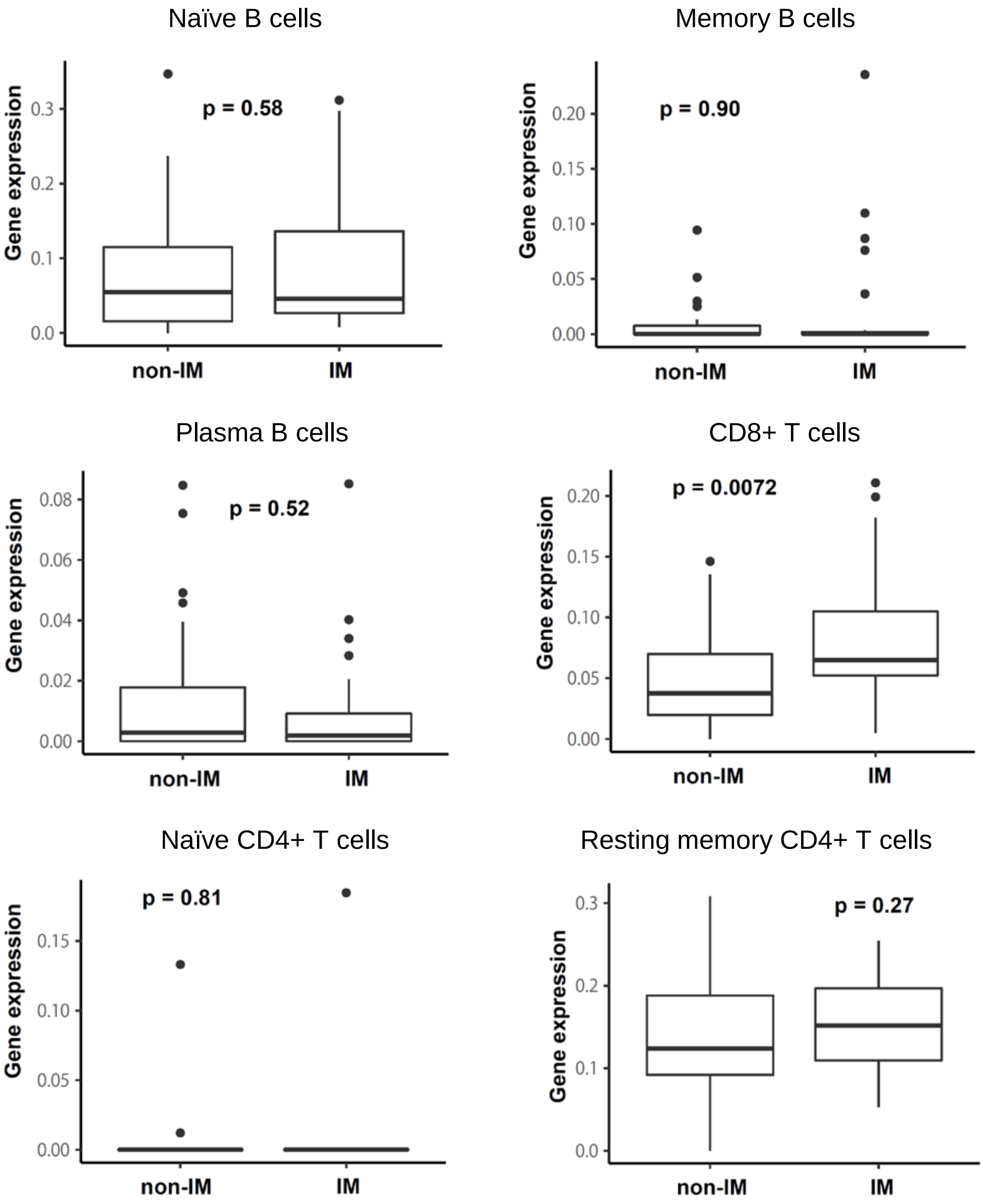
Naïve B cells
p = 0.58
Gene expression
0.0
0.1
0.2
0.3
non-IM
IM
Memory B cells
p = 0.90
Gene expression
0.00
0.05
0.10
0.15
0.20
non-IM
IM
Plasma B cells
p = 0.52
Gene expression
0.00
0.02
0.04
0.06
0.08
non-IM
IM
CD8+ T cells
p = 0.0072
Gene expression
0.00
0.05
0.10
0.15
0.20
non-IM
IM
Naïve CD4+ T cells
p = 0.81
Gene expression
0.00
0.05
0.10
0.15
non-IM
IM
Resting memory CD4+ T cells
p = 0.27
Gene expression
0.0
0.1
0.2
0.3
non-IM
IM

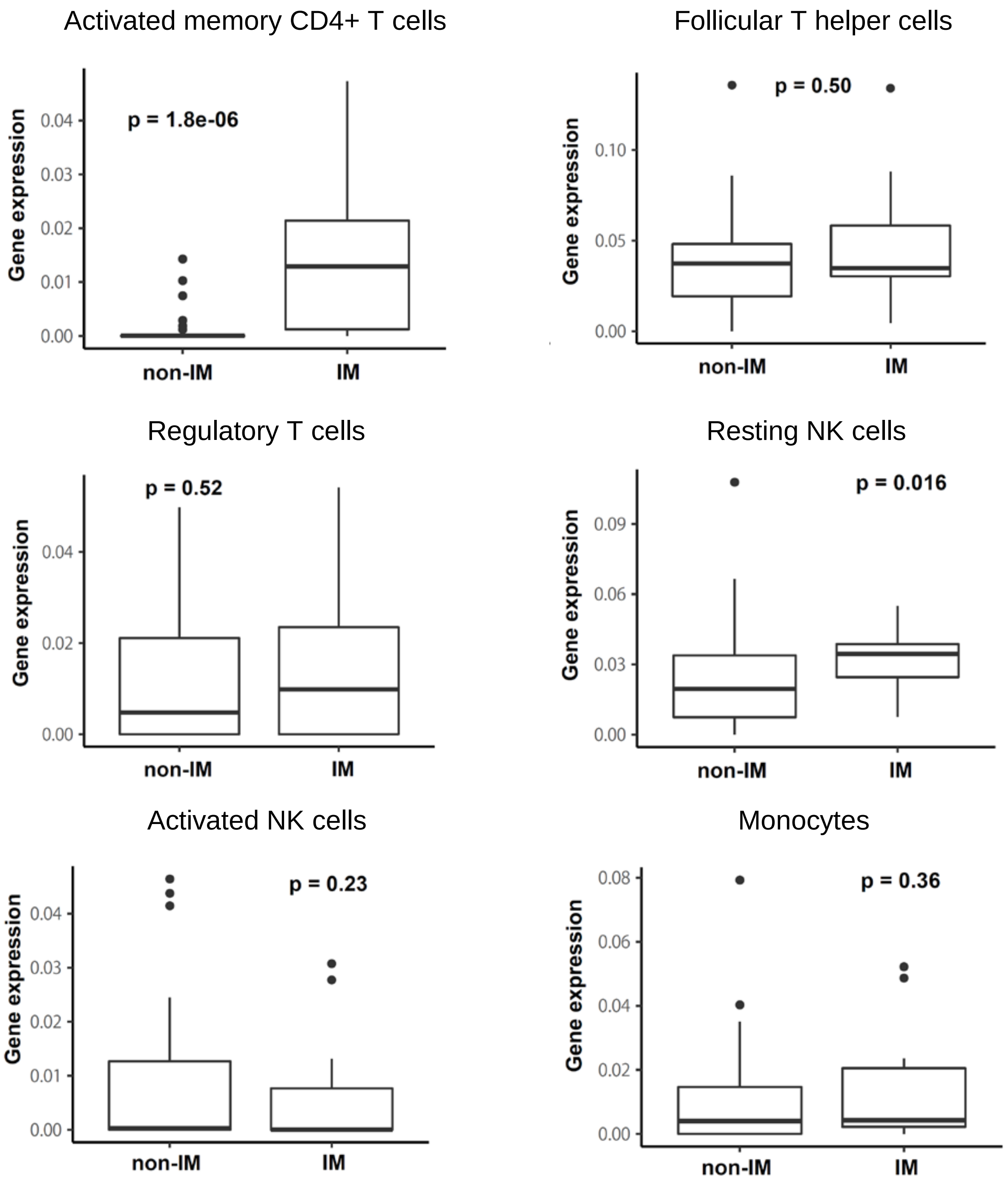
Activated memory CD4+ T cells
p = 1.8e-06
Gene expression
0.00
0.01
0.02
0.03
0.04
non-IM
IM
Follicular T helper cells
p = 0.50
Gene expression
0.00
0.05
0.10
non-IM
IM
Regulatory T cells
p = 0.52
Gene expression
0.00
0.02
0.04
non-IM
IM
Resting NK cells
p = 0.016
Gene expression
0.00
0.03
0.06
0.09
non-IM
IM
Activated NK cells
p = 0.23
Gene expression
0.00
0.01
0.02
0.03
0.04
non-IM
IM
Monocytes
p = 0.36
Gene expression
0.00
0.02
0.04
0.06
0.08
non-IM
IM

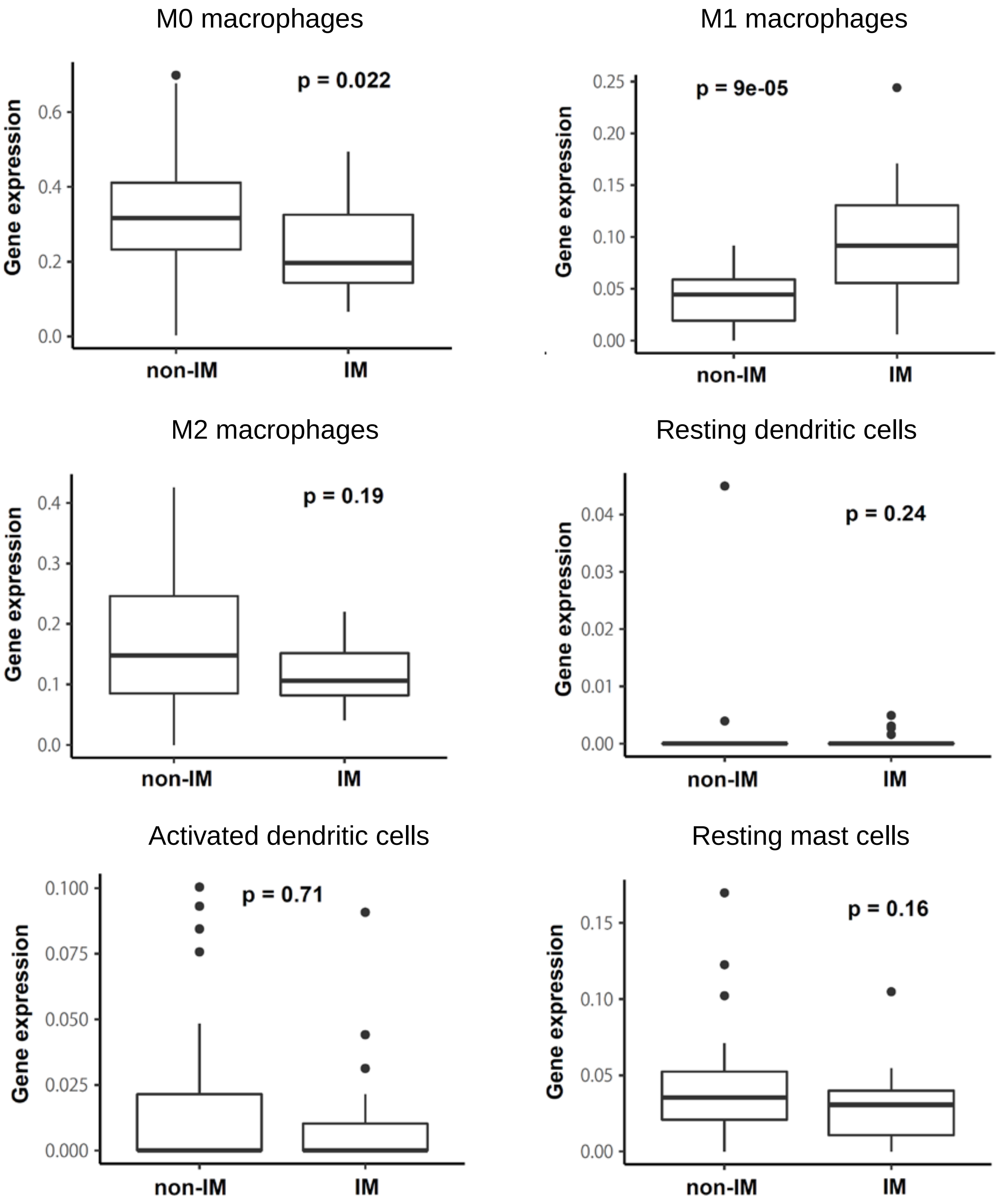
M0 macrophages
p = 0.022
Gene expression
0.0
0.2
0.4
0.6
non-IM
IM
M1 macrophages
p = 9e-05
Gene expression
0.00
0.05
0.10
0.15
0.20
0.25
non-IM
IM
M2 macrophages
p = 0.19
Gene expression
0.0
0.1
0.2
0.3
0.4
non-IM
IM
Resting dendritic cells
p = 0.24
Gene expression
0.00
0.01
0.02
0.03
0.04
non-IM
IM
Activated dendritic cells
p = 0.71
Gene expression
0.000
0.025
0.050
0.075
0.100
non-IM
IM
Resting mast cells
p = 0.16
Gene expression
0.00
0.05
0.10
0.15
non-IM
IM

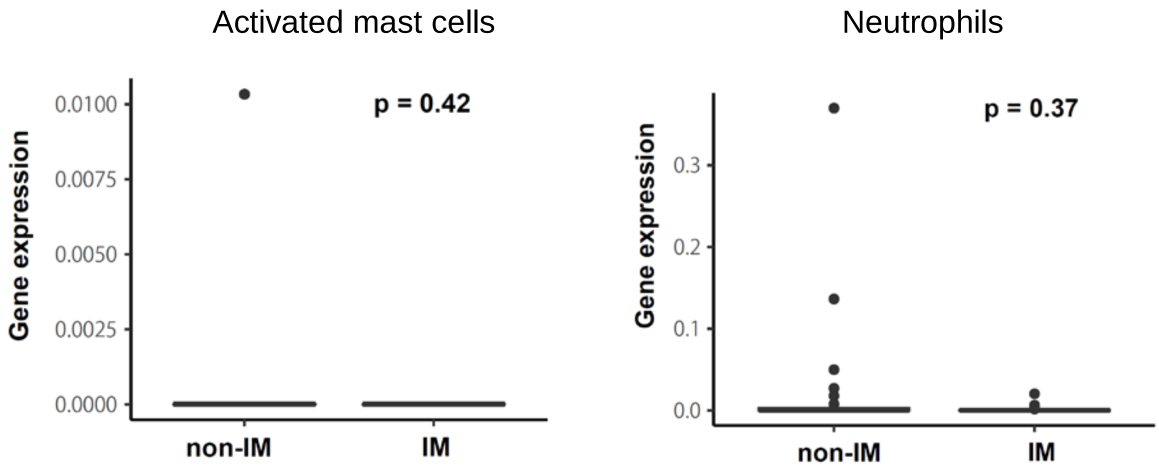

Figure S3. Gene expression of immune cell populations in the tumor microenvironment in patients with primary triple-negative breast cancer positive (immunomodulatory; IM) and negative (non-IM) for the 27-gene IO signature by CIBERSORT algorithm.

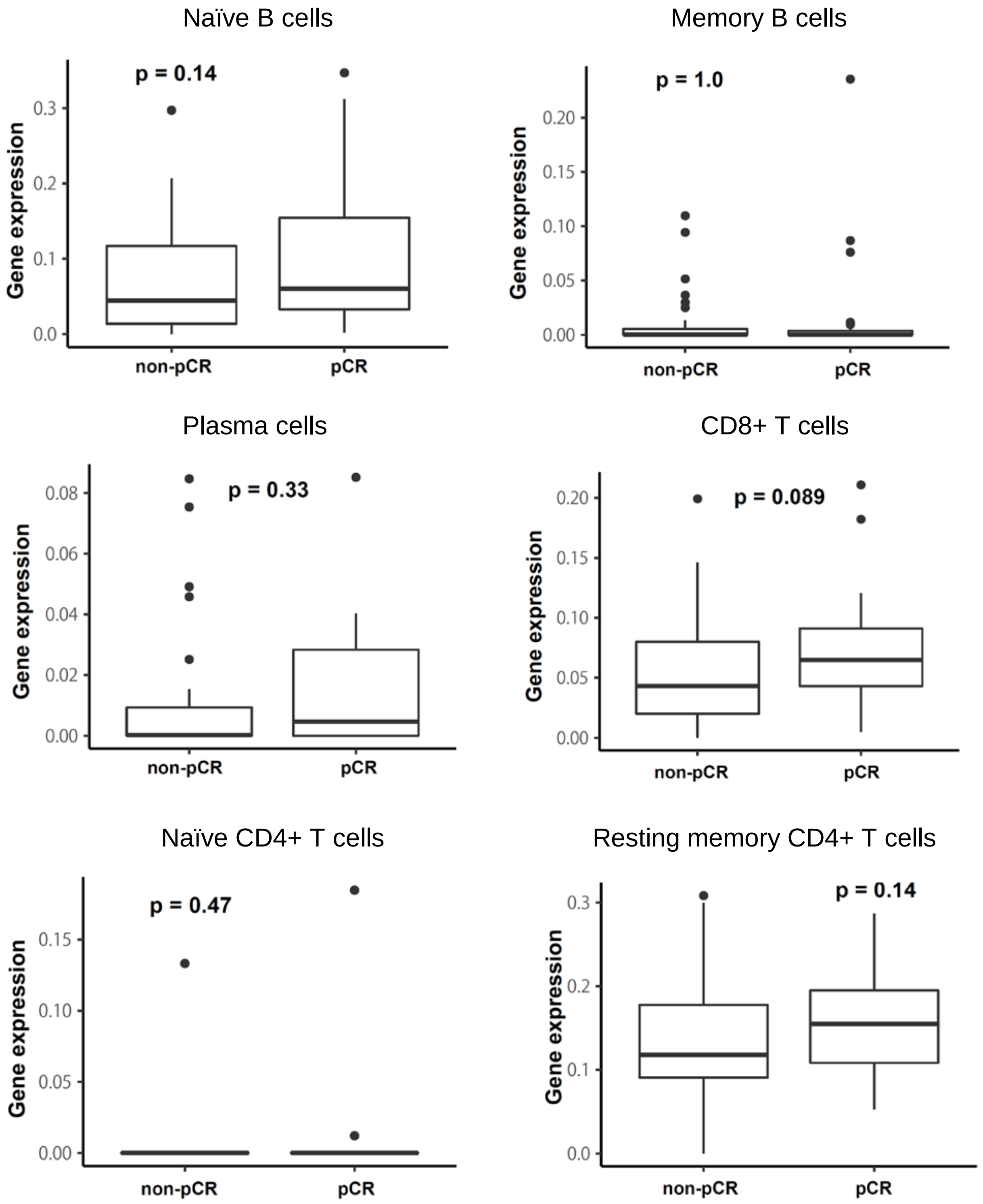
Naïve B cells
p = 0.14
Gene expression
0.0
0.1
0.2
0.3
non-pCR
pCR
Memory B cells
p = 1.0
Gene expression
0.00
0.05
0.10
0.15
0.20
non-pCR
pCR
Plasma cells
p = 0.33
Gene expression
0.00
0.02
0.04
0.06
0.08
non-pCR
pCR
CD8+ T cells
p = 0.089
Gene expression
0.00
0.05
0.10
0.15
0.20
non-pCR
pCR
Naïve CD4+ T cells
p = 0.47
Gene expression
0.00
0.05
0.10
0.15
non-pCR
pCR
Resting memory CD4+ T cells
p = 0.14
Gene expression
0.0
0.1
0.2
0.3
non-pCR
pCR

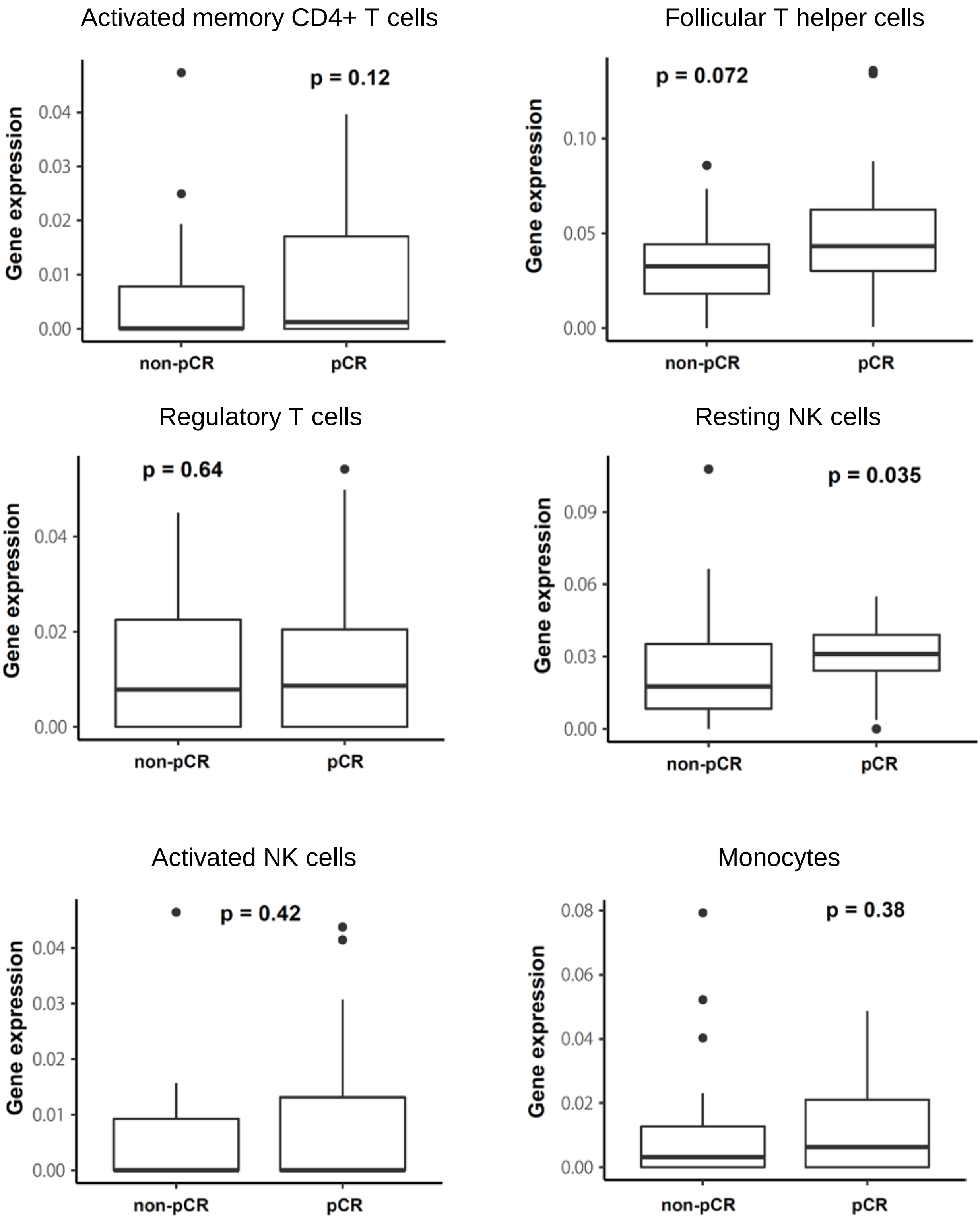
Activated memory CD4+ T cells
p = 0.12
Gene expression
0.04
0.03
0.02
0.01
0.00
non-pCR
pCR
Follicular T helper cells
p = 0.072
Gene expression
0.10
0.05
0.00
non-pCR
pCR
Regulatory T cells
p = 0.64
Gene expression
0.04
0.02
0.00
non-pCR
pCR
Resting NK cells
p = 0.035
Gene expression
0.09
0.06
0.03
0.00
non-pCR
pCR
Activated NK cells
p = 0.42
Gene expression
0.04
0.03
0.02
0.01
0.00
non-pCR
pCR
Monocytes
p = 0.38
Gene expression
0.08
0.06
0.04
0.02
0.00
non-pCR
pCR

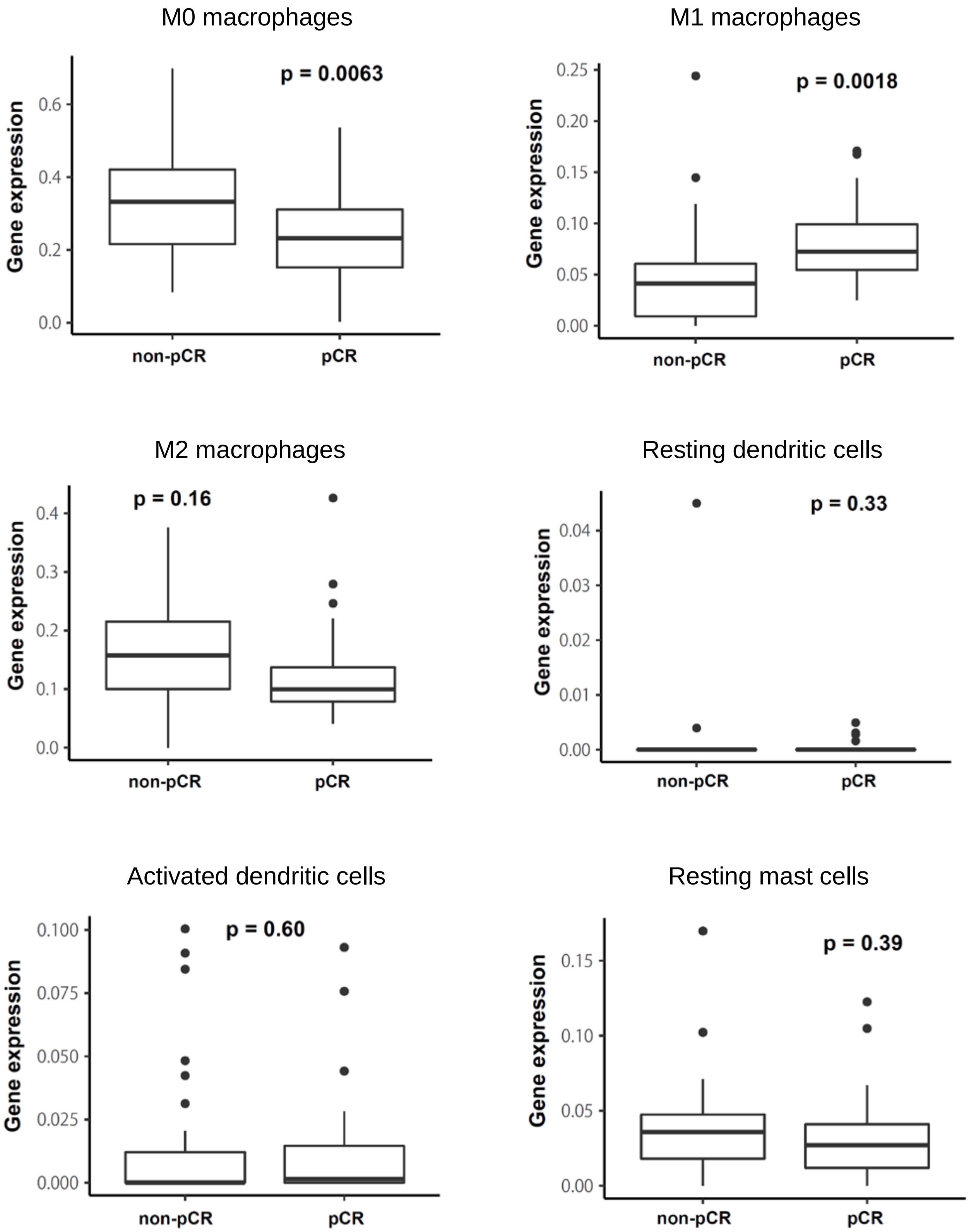
M0 macrophages
p = 0.0063
Gene expression
0.0
0.2
0.4
0.6
non-pCR
pCR
M1 macrophages
p = 0.0018
Gene expression
0.00
0.05
0.10
0.15
0.20
0.25
non-pCR
pCR
M2 macrophages
p = 0.16
Gene expression
0.0
0.1
0.2
0.3
0.4
non-pCR
pCR
Resting dendritic cells
p = 0.33
Gene expression
0.00
0.01
0.02
0.03
0.04
non-pCR
pCR
Activated dendritic cells
p = 0.60
Gene expression
0.000
0.025
0.050
0.075
0.100
non-pCR
pCR
Resting mast cells
p = 0.39
Gene expression
0.00
0.05
0.10
0.15
non-pCR
pCR

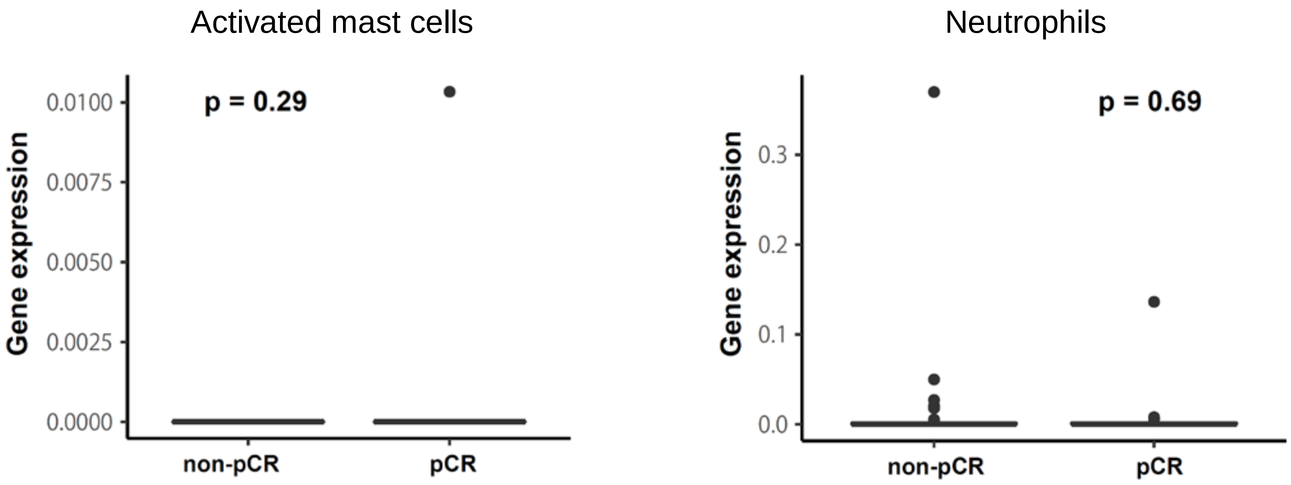

Figure S4. Gene expression of immune cell populations in the tumor microenvironment in patients with a pathological complete response (pCR) or without a pCR (non-pCR) by CIBERSORT algorithm.

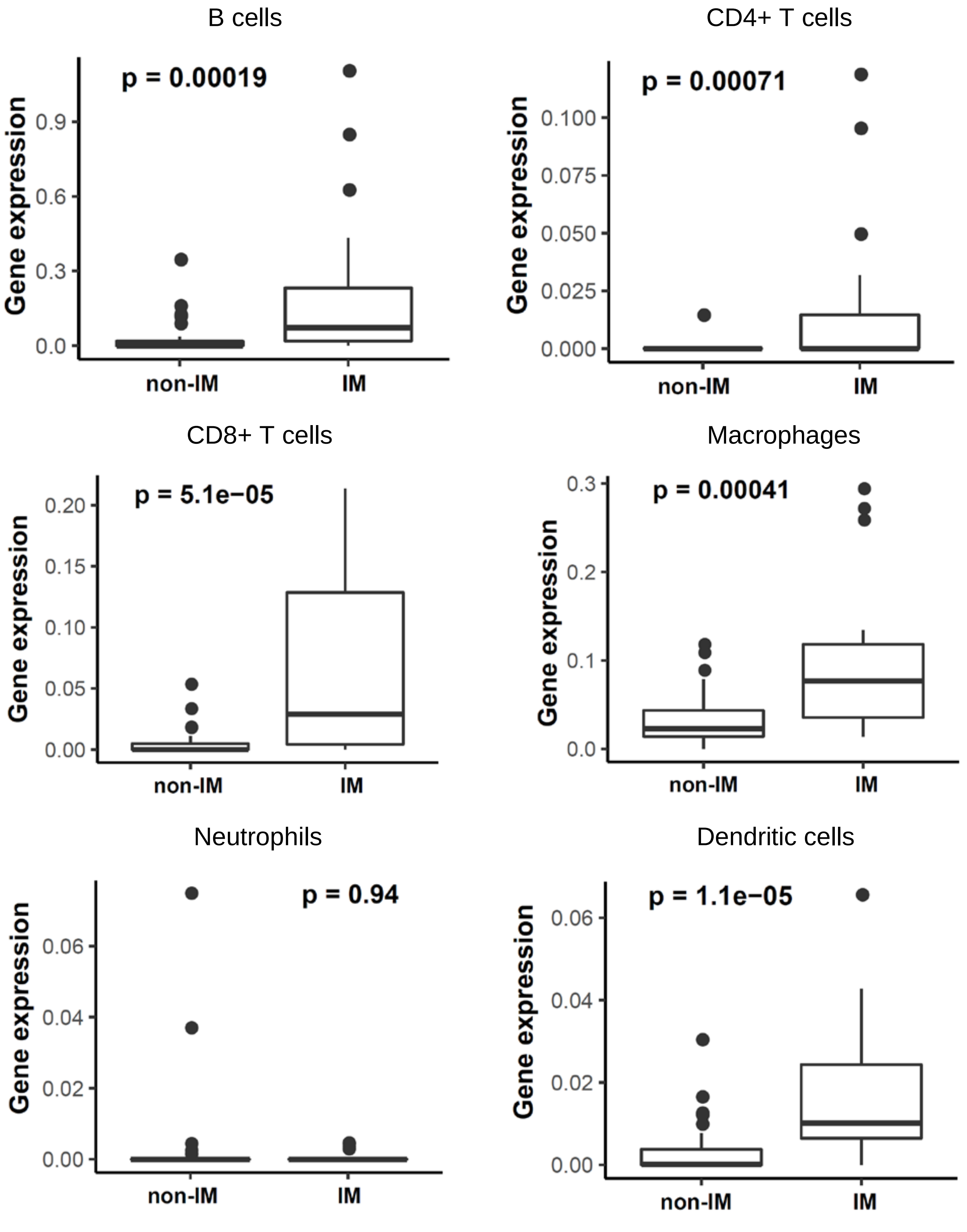

Figure S5. Gene expression of immune cell populations in the tumor microenvironment in patients with primary triple-negative breast cancer positive (immunomodulatory; IM) and negative (non-IM) for the 27-gene IO signature by xCell algorithm.

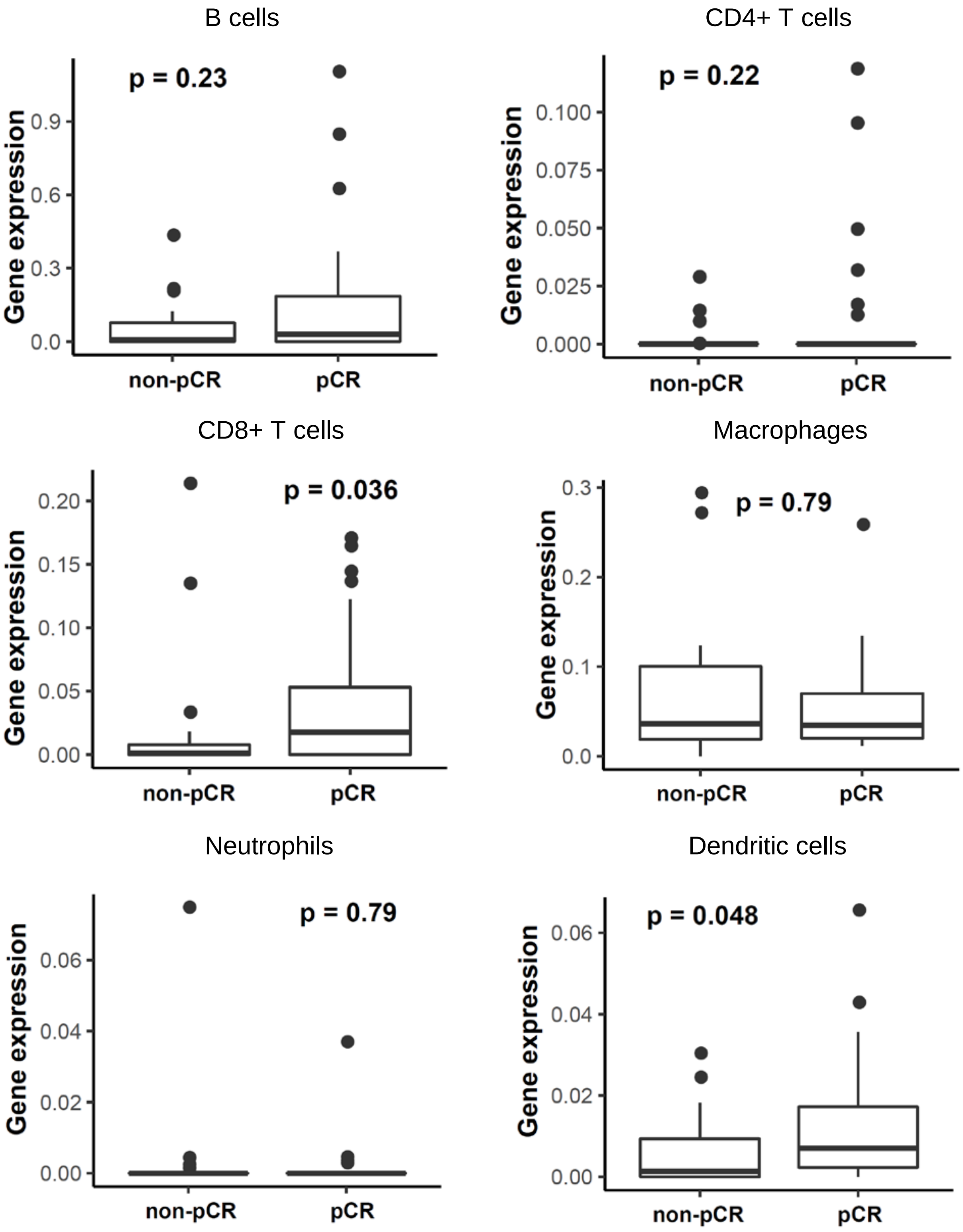

Figure S6. Gene expression of immune cell populations in the tumor microenvironment in patients with a pathological complete response (pCR) or without a pCR (non-pCR) by xCell algorithm.
